# Cases of Tic Douloureux, and Other Forms of Neuralgia

**Published:** 1834-10-01

**Authors:** 


*Cases of Tic Douloureux, and other Forms of Neuralgia.* By JOHN SCOTT, Surgeon to the London Hospital, &c.—London, 1834. 8vo. pp. 52.

MR. SCOTT is of opinion that the constitutional causes of tic douloureux may be arranged under five heads:

"1. A plethoric state of system, which, however, it must be observed, very rarely gives rise to the affection.

"2. An asthenic state of system, which certainly produces the disease much more frequently than the opposite condition; hence its frequent occurrence in the decline of life, when the power of the circulation begins to fail, and during periods of mental suffering and anxiety.

"3. A gouty or rheumatic diathesis seems particularly liable to the disease, which often occurs in conjunction with affections of this character in other textures, or immediately on their subsidence; and it is observed that persons who are habitually exposed to cold and moisture, as fishermen, and the inhabitants of marshy districts, are particularly liable to the disease in question.

"4. A disordered condition of the digestive organs, attended by the usual symptoms which characterise such derangement.

"5. The impression of malaria on the system; in which case the disease assumes the intermittent character, the paroxysms recurring regularly at a given hour, and increasing or lessening in duration as the disease becomes more or less severe." (P. 17.)

We doubt whether a sufficient number of cases of this formidable disease have yet been observed and registered, to allow us to generalize on the classes of men most subject to it; but we doubt still more whether, when this shall have been done, fishing will be found to be among the predisposing causes. It is not among fishermen that we should look for cases, but among those who are hypochondriacal by profession; such as clerks, comic actors, authors, and reviewers.

In illustration of the first variety, our author says,

"When the disease depends on a plethoric state of system, it will of course be attended with the symptoms which usually characterise that condition.

"A gentleman, about fifty years of age, of a full habit of body, affected with constant pain in the head, and occasional dizziness and vertigo, laboured under disease of the supra-orbitar branch of the fifth pair of nerves for some years, which resisted all the various modes of treatment that were adopted. At length he was affected with an apoplectic seizure, for the relief of which he was largely bled, and the antiphlogistic treatment carried to its fullest extent; in consequence of this discipline, he not only recovered from the attack, but the tic douloureux also disappeared, and he remained free from the disease for many years." (P. 18.)

On the other hand, when the disease depends on debility, the carbonate of iron, recommended by Mr. Copland Hutchison, often effects a cure.

"A young lady, twenty-three years of age, of a delicate scrofulous habit, whom I had previously attended on account of a disease in the hip-joint, applied to me on the 24th of February, labouring under a severe attack of tic douloureux, chiefly affecting the infra-orbitar branch of the fifth pair of nerves on the right side of the face: the pain extended during the paroxysms to the ear, the temple, the forehead, the lower jaw, and the lips, and terminated abruptly at the mesial line of the face. She stated, that on Friday, the 14th of February, she was first attacked with the disease in the parts above described, being suddenly seized with a violent shooting pain, as if a sharp instrument were darting through the face; and this had been preceded by slight uneasiness in the parts for about a quarter of an hour: the acute pain lasted for about an hour, and then suddenly ceased, the uneasiness by which it had been preceded subsiding in about half an hour afterwards. During the continuance of the pain, the part was so exquisitely sensitive, that she could not bear the slightest touch; but she thought she experienced some relief from her agony by powerfully compressing it with her hand. Every day, from the time above mentioned, she had experienced three or four attacks similar to that I have just described, with occasional slight variations in their severity and duration; generally they occurred spontaneously, and sometimes they appeared to be brought on by mastication or talking.

"She was directed to take a drachm of the carbonate of iron every four hours, to be much in the open air, and to use a generous diet.

"Under this treatment the paroxysms soon became less severe, less frequent, and of shorter duration, until the 3d of March, on which day she had a very severe attack; after which they entirely ceased until the beginning of April, when she had one or two slight attacks, but not of any severity or duration. Her health was much improved by the treatment employed, and she has not had any return of the affection." (P. 19.)

The next case is one of a delicate woman, who was cured of her first attack by the carbonate of iron, taken for a month; and on a relapse occurring, by the sulphate of quinine. This patient used the veratria without benefit, though it produced so much pain that she could scarcely endure its application.

Mr. Scott thinks that a gouty or rheumatic diathesis is the most frequent among the constitutional causes of neuralgia; and he gives, as an illustration, the following instance, in which the disease was seated in the first branch of the fifth pair of nerves on the right side.

"The pain commenced with a falling of the eyelid and corrugation of the brow, attended with a throbbing sensation in the part, which symptoms indicated the approach of the attack. It came on gradually, commencing at sunrise; became very severe about ten o'clock in the morning; and at noon the pain would be often so excruciating, that he could neither sit, stand, nor walk, but actually writhed in agonies on the carpet, and was obliged to go to bed to hide his suffering; from noon, the pain gradually decreased in severity until the evening, when it entirely subsided; so that he slept perfectly well during the early part of the night, and was awoke in the morning by the renewal of his sufferings. The attacks were more violent in cold, particularly during an easterly wind, than in warm weather; but they always affected the same spot, occurred at the time and in the manner above described, and pursued their own course, without being relieved by any remedies, or aggravated by any exciting causes: neither mastication nor talking would produce them, nor increase their severity; but at one time they certainly appeared to be much more violent during a period of great mental anxiety. These attacks would recur daily for two or three weeks, and then spontaneously subside for two or three months, and again recur without any assignable cause; at other times they would occur regularly for a day or two, and then there would be an interval of a week or more before any fresh attack: in short, there was no regularity in the frequency of their recurrence, nor in the length of time they would continue; but he never passed a year without many attacks, for a period of twenty-eight years, with the exception of three successive years, during the whole of which he was subject to constant attacks of acute rheumatism, in almost every joint in the body in succession, and they were so severe, of such continuance, and so frequently renewed, that he scarcely left the house during that period; but he was perfectly free from tic douloureux the whole of this time. As soon, however, as the rheumatism disappeared, the original disease was reproduced, with its usual frequency and severity. Of course, a vast variety of treatment was adopted at various times: among other remedies, he took large quantities of quinine of iron and of colchicum, but nothing appeared to have the slightest influence on the disease; blisters, leeches, opiates, &c. were employed, and he was advised to have the nerve divided, but would not consent to the operation." (P. 22.)

He was cured by sarsaparilla and purgatives.

The fifth variety is that in which the disease is intermittent. The patient, whose case is given as an example of this form, was a lady who had two attacks daily, one at two A.M., and the other at three P.M. The sulphate of quinine, the carbonate of iron, and bark, with muriate of ammonia, were given without benefit, but she was cured by large doses of the liquor arsenicalis: ten minims were given at first every six hours, and afterwards every two hours.

So much for the treatment when the disease is constitutional. In protracted cases, however, the disease becomes local, and the remedies hitherto mentioned are found to fail. In such cases, Mr. Scott, some years ago, tried the effects of a topical application. "The plan I adopted was to keep constantly applied to the part, on a piece of flannel, an ointment composed of one drachm of tartarised antimony and an ounce of mercurial ointment, renewing it as frequently as it could be borne; the object being to produce such a degree of irritation on the skin as would insure the mercurial influence on the part." (P. 31.) This ointment, and some aperient pills, removed the neuralgia of a patient aged sixty-two, who had suffered at intervals for thirteen years, and who had gone through the ordinary routine of remedies without advantage.

Our author then thought that the iodurets of mercury might prove more effectual, and accordingly tried an ointment composed of the deuto-ioduret (℈ij. to ℥i. of lard,) in the case of a lady who had gone through not only the most violent attacks of the disease, but the following remedies:

"Sulphate of quinine, fifteen grains in the day.

"Carbonate of iron, three ounces in the day for many weeks.

"Arsenic, till the gastric irritation it occasioned prohibited its continuance.

"Belladonna, till the sensorium was seriously affected by it.

"Musk, black drop, hyoscyamus, colchicum, and mercury in small doses, until the mouth became affected." (P. 35.)

She was cured in six weeks.

Mr. Hollis, of Lewisham, has furnished our author with a case, in which the same remedy proved successful, though the patient was seventy-three years of age.

The rest of the cases we will give in a tabular form, to save room.

| Patient. | Situation of Disease. | Remedies employed. | Result. |
|---|---|---|---|
| A lady in Albemarle-st. ill eight years ......... | The supra-orbitar branch of the 5th pair on the left side ............... | Deuto-ioduret & Proto-ioduret of Mercury, Mercurial Ointment, and Purgatives. | Cured. |
| A gentleman, aged 92 | The supra-orbitar and infra-orbitar branches of the fifth pair ......... | Deuto-ioduret of Mercury ..................... | Cured. |
| A young woman, aged 21.............................. | The left breast ... | Proto-iod. of Mercury, and supporting the breast by a bandage. | Cured. |
| A young lady ............ | The left knee ..... | Proto-ioduret of Mercury ..................... | Cured? |

Our author then says, "It is unnecessary to increase the length of the foregoing list of cases, since my object is not to display the marvellous effects of any specific remedy, in controlling the painful disease which has been the subject of the preceding pages, but to analyse the constitutional causes of the affection." (P. 50.)

We cry you mercy, Mr. Scott! We thought that your object had been "to display," &c.; for you tell us of none but successful cases. When a man sets about analysing the causes, or the treatment of a disease, we expect him to make a clean breast, and give us an account of his failures: the painter, whose office is to select merely the agreeable, may delight us with the bright skies and rich verdure of a tropical climate; but the faithful chronicler must speak also of poisonous serpents and overwhelming tornadoes. Mr. D'Israeli, however, tells us that many authors consider the first edition of a work as a sketch merely, to be distributed among their friends, by whose judgment they are to be guided in expanding the outline into a book. We hope therefore that the sale of this treatise may be so rapid as to enable Mr. Scott immediately to supply this deficiency. Meanwhile we bid him farewell, recommending his new remedy for trial, and his essay for perusal.

